# Modeling of Novel Diagnostic Strategies for Active Tuberculosis – A Systematic Review: Current Practices and Recommendations

**DOI:** 10.1371/journal.pone.0110558

**Published:** 2014-10-23

**Authors:** Alice Zwerling, Richard G. White, Anna Vassall, Ted Cohen, David W. Dowdy, Rein M. G. J. Houben

**Affiliations:** 1 Department of Epidemiology, Johns Hopkins Bloomberg School of Public Health, Baltimore, Maryland, United States of America; 2 TB Modelling Group, TB Centre, London School of Hygiene and Tropical Medicine, London, United Kingdom; 3 Department of Global Health and Development, London School of Hygiene and Tropical Medicine, London, United Kingdom; 4 Department of Epidemiology, Harvard School of Public Health, Boston, Massachusetts, United States of America; University of Erlangen-Nuremberg, Germany

## Abstract

**Introduction:**

The field of diagnostics for active tuberculosis (TB) is rapidly developing. TB diagnostic modeling can help to inform policy makers and support complicated decisions on diagnostic strategy, with important budgetary implications. Demand for TB diagnostic modeling is likely to increase, and an evaluation of current practice is important. We aimed to systematically review all studies employing mathematical modeling to evaluate cost-effectiveness or epidemiological impact of novel diagnostic strategies for active TB.

**Methods:**

Pubmed, personal libraries and reference lists were searched to identify eligible papers. We extracted data on a wide variety of model structure, parameter choices, sensitivity analyses and study conclusions, which were discussed during a meeting of content experts.

**Results & Discussion:**

From 5619 records a total of 36 papers were included in the analysis. Sixteen papers included population impact/transmission modeling, 5 were health systems models, and 24 included estimates of cost-effectiveness. Transmission and health systems models included specific structure to explore the importance of the diagnostic pathway (n = 4), key determinants of diagnostic delay (n = 5), operational context (n = 5), and the pre-diagnostic infectious period (n = 1). The majority of models implemented sensitivity analysis, although only 18 studies described multi-way sensitivity analysis of more than 2 parameters simultaneously. Among the models used to make cost-effectiveness estimates, most frequent diagnostic assays studied included Xpert MTB/RIF (n = 7), and alternative nucleic acid amplification tests (NAATs) (n = 4). Most (n = 16) of the cost-effectiveness models compared new assays to an existing baseline and generated an incremental cost-effectiveness ratio (ICER).

**Conclusion:**

Although models have addressed a small number of important issues, many decisions regarding implementation of TB diagnostics are being made without the full benefits of insight from mathematical models. Further models are needed that address a wider array of diagnostic and epidemiological settings, that explore the inherent uncertainty of models and that include additional epidemiological data on transmission implications of false-negative diagnosis and the pre-diagnostic period.

## Introduction

The last decade has seen a dramatic increase in the development of novel diagnostic tests for active tuberculosis (TB) [1]–[4]. As a result policy makers must decide what novel diagnostics to implement with the limited resources available to them. To date the contribution of modeling to decision-making processes has been limited; despite the rapid growth of interest in TB diagnostics, in many cases models are not available to aid decision-making, particularly within country. As policy makers face questions about implementation of novel diagnostic strategies with substantial budgetary implications, the demand for these models will increase. Given the wide variety in modeling approaches, methods and objectives, both those developing and those using models can benefit from a synthesis and evaluation of current TB diagnostic modeling practices.

The development of novel diagnostics is not limited to technological advancements, but includes novel diagnostic algorithms and systems of diagnosis that allow novel or existing diagnostic tools to affect changes in clinical practice leading to improved patient outcomes[1]–[4]. Novel tests may boast a range of improved attributes. However the impact of any novel test depends on how that test is implemented and for what intended population. Novel tools cannot benefit TB patients unless they lead to earlier or more appropriate initiation of treatment. Likewise, evaluations of novel tests should account for the role of empiric therapy as individuals may still be treated in the absence of a positive test result, and this can have important implications on the impact of novel tools. New technologies are often (although not always) accompanied by increased costs, relating to the test itself and/or the systems into which they are implemented. In the face of multiple technological options and even more potential algorithms and implementation plans, deciding which novel diagnostic(s) to implement, and how, is a very complex process [5]. Such decisions must involve careful consideration of available strategies, requiring an understanding of the potential costs, population level impact for each test upon implementation in a given setting and uncertainty around these factors. Mathematical modeling, including transmission modeling, health systems modeling and economic evaluation can help to make these decision-making processes more transparent and data-driven; in the absence of such models, it is difficult to utilize existing data in a systematic fashion for decision-making.

The objectives of this paper were to systematically review all studies employing mathematical modeling to explore the potential epidemiological impact and/or cost-effectiveness of novel diagnostic tests for TB and to identify and discuss key methodological challenges and limitations of existing models and gaps in empirical evidence required to populate such models. This review was undertaken within the context of the TB Modelling and Analysis Consortium (TB MAC) meeting on modeling of novel TB diagnostics, where preliminary results were discussed.

## Methods

We carried out a systematic literature review to identify existing TB modeling papers that evaluated novel tools or algorithms to diagnose active TB. Our focus was to identify models exploring novel diagnostic technology or models concerned with understanding the TB diagnostic pathway. Models that explored the impact of alternative case finding approaches not related to new diagnostic tools (e.g. active versus passive case finding) were not included.

### Search strategy

The medical literature was searched for relevant studies in PUBMED; the detailed search string is included in the supplementary material. Personal libraries of TB MAC members were searched for modeling papers (free text search for ‘model’), and mathematical modeling journals were scanned for any papers describing TB models. Within this set of retrieved citations, potentially relevant records were identified through a text search for ‘diagn’ OR ‘test’ OR ‘screen’. Citations of all selected original articles [6] published after the 1^st^ of January 2000 to March 1^st^ 2013, were reviewed. The database of relevant citations is regularly updated by TB MAC and available online at: http://www.tb-mac.org/Resources/.

### Inclusion and exclusion criteria

Papers were eligible for inclusion if they were written in English and published after the 1^st^ of January 2000 to restrict to recent diagnostic strategies with relevance to current policy decisions. Papers were excluded if the model focused on diagnosing latent TB infection, or evaluated only diagnostic tools that fell within the existing standard of care at the time of publication, based on our understanding of TB diagnostic practices. Therefore this review was focused on modelling novel diagnostic tools or algorithms for active TB that are NOT currently in use as the standard of care. For the purposes of this review, the Xpert MTB/RIF test (Xpert, Cepheid Inc., CA, USA) was not yet considered as standard of care in any setting. The selected papers were independently assessed by two reviewers (AZ and RMGJH) for inclusion and all included studies underwent double data extraction by the same two reviewers; disagreements were resolved through consensus. In defining ‘mathematical model’ we followed *Garnett et al* [7] and included decision analytic, transmission, operational or within-host models, but excluded purely statistical models and studies using models to only estimate resource requirements without corresponding measures of health-related outcomes [8].

Papers were grouped into three subject areas that were not mutually exclusive: 1) population level impact/transmission, defined as models including a transmission component or measuring epidemiological impact at the population level; 2) health systems, defined as models including compartments that represent points of interactions between patient and health care providers or institutions; and 3) model based cost-effectiveness and cost-utility analyses, following the definitions by Drummond et al. [8]. To further clarify these definitions, transmission modeling generally assesses the population level impact of interventions in terms of changes in TB incidence, TB prevalence or TB mortality over time and can estimate the potential future benefit of introducing a novel diagnostic intervention on those population-level outcomes. Health systems models, which may include transmission and economic evaluations, involve explicitly modeling the interaction between patients and the health system or health care provider. These models are important for evaluating TB diagnostic interventions in the local context and can improve our understanding of operational elements such as patient and diagnostic delay or other key elements of the patient–provider interaction that may influence a test's potential impact and costs. We followed the definitions of Drummond et al. for cost-effectiveness and cost-utility analyses, including any type of model (transmission, health system, cohort, etc.) that includes a comparison of two or more alternative strategies both in terms of costs and consequences (efficacy or effectiveness) [8]. Economic evaluations can rely on many different types of models, but economic evaluations are defined by the study purpose or question they are answering (cost-effectiveness, budget impact, etc.), using different types of models to answer different economic questions. Decision analysis and Markov models are the most common approach for evaluating cost-effectiveness of novel diagnostics; however cost components can also be added to transmission or health systems models.

### Data extraction and synthesis

For all papers information was extracted pertaining to the research question and result. Extracted data included: details of the population and diagnostics pathways that were explored, model methodology (model type, sensitivity analyses, details of model structure) and, where appropriate, details on how the cost-effectiveness analysis was conducted, including questions about the costing perspective employed and scope of the costing parameters. A complete summary of all data extracted is available in the supplementary material. The results were discussed by a broad group of experts during the TB MAC meeting on “Impact and cost-effectiveness of current and future diagnostics for TB” in Amsterdam, April 24–25^th^ 2013 (www.tb-mac.org/WorkAreas/WorkArea/4).

## Results and Discussion

**Figure 1:**
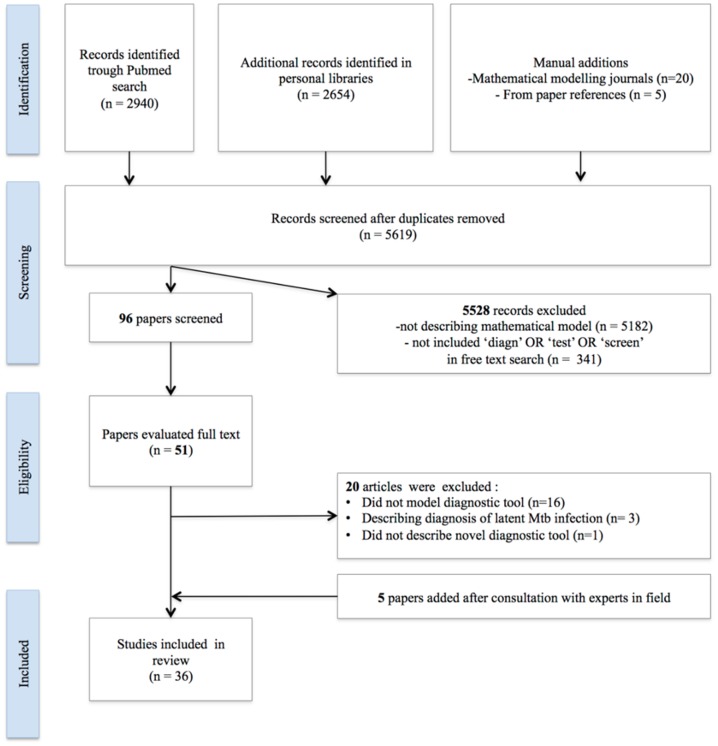
Systematic review flowchart for selection of papers.

Out of 5619 unique records that were found in the literature search, 91 records were selected for evaluation, of which 46 underwent full-text evaluation. After full-text screening 31 papers were selected. Consultation with experts in the field yielded five additional papers; therefore 36 papers were included for data extraction (see Figure 1). The complete summary of all included papers and extracted data is available in Table S1.1 thru Table S3.4 in Data S1.

### Is enough modeling being done?

Since the approval of Xpert in 2010, a sharp increase in TB diagnostic modeling has been seen both among papers modeling Xpert and among papers evaluating alternative nucleic acid amplification tests (NAATs). Compared with only 8 TB modeling papers published from 2000–2007 inclusive (approximately one per year), 10 TB diagnostic modeling papers were published in 2012, 6 of which focused on Xpert.

The 36 included papers were grouped into the three non-exclusive categories: 16 papers included population impact/transmission modeling, 5 were health systems models, and 24 included cost-effectiveness models. Novel tests evaluated across the 35 papers included: several NAATs, light-emitting diode (LED) microscopy, Determine TB-LAM (Alere Inc., MA, USA) a lateral-flow urine lipoarabinomannan detection assay, FastPlaque TB and FastPlaque-Response (Biotec Labs Ltd, UK), serological tests including Anda TB (Anda Biologicals, Strasbourg), MTT assay, a non-commercial colorimetric assay (ICN Biomedicals) and sputum processing methods including bleach sedimentation and sample dilution. A table of novel TB diagnostic tests included in the evaluated studies with brief descriptions is included in Table 1. Novel diagnostic strategies included the expansion of culture, drug sensitivity testing (DST), chest x-ray and mass miniature radiography (MMR). Several hypothetical diagnostic tests, scenarios or algorithms were also evaluated including: hypothetical NAATs with improved speed, sensitivity and specificity; a dipstick style test; rapid DST; tests resulting in reduction in diagnostic delay; and rapid point of care with improved sensitivity, specificity and reduced time to results compared to smear microscopy or conventional drug susceptibility testing (Tables S1.2, S2.2 & S3.3 in Data S1).

**Table 1:** Novel diagnostic tests for active TB disease evaluated in included modeling review.

Novel diagnostic tests evaluated	Type of Diagnostic	Method	Sample type
**Nucleic Acid Amplification Tests (NAATs)**			
GeneXpert MTB/RIF (Xpert) (Cepheid, USA)	NAAT	Real-time PCR based technique to detect both presence of MTB and RIF resistance using an automated cartridge based design	Resp. spec.
INNO-LiPA Rif. TB (Immunogenetics, Belgium)	Line probe	A reverse hybridization-based line probe assay that detects mutations is the rpoB “hotspot” gene region (RIF resistance)	Resp. spec. and/or liquid/solid culture
MTBDRplus (Hain Lifescience, Germany)	Line-probe	Molecular genetic assay for identification of resistance to rifampicin and/or isoniazid. Mycobacterial DNA is extracted from the specimen, specifically amplified via PCR and detected on a membrane strip using reverse hybridization and an enzymatic color reaction.	Resp. spec. and/or liquid/solid culture samples
GenProbe Amplified Mycobacterium Tuberculosis Direct Tests (MTD) (Gen-Probe Inc., USA)	NAAT	Transcription mediated amplification of rRNA, detects Mycobacterium tuberculosis rRNA directly and rapidly	Resp. spec.
Cobas Amplicor (Roche Diagnostics)	NAAT	PCR amplification of 16s rRNA	Resp. spec.
**Non-NAAT based tests**			
LED Microscopy	Microscopy	Light Emitting Diode (LED) based fluorescence microscope, energy-efficient, does not require a dark room	Resp. spec.
FastPlaque-Response (Biotech, UK)	Phage-based assay	Allows for detection of Rifampicin resistance; Phage-based assay that detects live MTB in a plaque assay in a lawn of rapidly growing detector cells. Samples are incubated with and without RIF overnight	Resp. spec.
FastPlaque TB (Biotech, UK)	Phage-based assay	Mycobacteriophage (virus that infect TB) - Phage-based assay that detects live MTB in a plaque assay in a lawn of rapidly growing detector cells.	Resp. spec.
MTT assay (ICN Biomedicals)	Colorimetic assay	Noncommercial colorimetric assay that uses an indicator of cellular growth and viability whose oxidized yellow form becomes purple after reduction to formazan by the dehydrogenases of live bacterial cells	Liquid/solid culture samples
Bleach sedimentation on sputum samples	Sputum processing to increase dx yield	Bleach digestion of sputum, followed by specimen concentration step such as sedimentation	Resp. spec.
Anda TB (***Anda Biologicals, Strasbourg***)	Serological ELISA test	ELISA based detection of antibodies elicited by antigens of MTB that are recognized by humoral immune system	Blood samples
Diluting sputum before MTD Gen-probe	Specimen processing	1∶10 dilution of the processed specimen	Resp. spec.
Determine TB-LAM (Alere, USA)	Lateral-flow immune-chromatographic assay	Lateral-flow immune-chromatographic assay detects lipoarabinomannan (LAM) – an immunogenic glycolipid in the cell way of MTB- in urine. First true POC test, excellent specificity and higher sensitivity than sputum smear microscopy in immuno-compromised adults	Urine samples

Abbreviations: MTB: Mycobacterium tuberculosis, RIF: rifampicin, PCR: polymerase chain reaction, POC: point-of care, ELISA: enzyme linked immune-sorbent assay, Resp. Spec.: respiratory specimens, dx: diagnostic.

The majority of studies (83%, 30/36) modelled high TB incidence settings; 39% (14/36) of evaluated studies modelled the South African setting specifically, reflecting the large burden of TB in South Africa and the recent move and investments towards novel technologies in improving TB control. Only 6/36 (17%) studies -all cost-effectiveness analyses- modelled low TB incidence settings including the United States, United Kingdom, and Finland. While the majority of studies are modelling settings with the highest TB burden, many important epidemiological settings (e.g., China, Southeast Asia outside of India) have few or no models to provide insight into potential cost-effectiveness or epidemiological impact of novel diagnostics or diagnostic strategies. As a result decisions with important repercussions for the TB epidemic and TB control budgets are frequently being made in the absence of supporting insight that models could provide. As tests and diagnostic algorithms are developed and evaluated, models are needed that represent a wide range of diagnostic tests, strategies and settings to help support data-driven policy decisions.

### What questions are modelling studies trying to answer?

Models covered a wide range of objectives in evaluating novel diagnostics. Transmission and health system models investigated the impact of diagnostic delay (n = 5), and demonstrated several key insights including a threshold for average delay to diagnosis beyond which an epidemic will escalate [9], the importance of applying novel diagnostics in both private and public health systems to reduce diagnostic delay [10], and that test sensitivity was a key determinant of diagnostic delay [11]. In prison populations it was shown screening at entry and annually with MMR could keep TB prevalence below 1% [12]. In the case of drug-resistant TB, it was demonstrated that current strategies with long delays to diagnosis will probably not halt the spread of MDR-TB, and reducing the delay through improved access to DST and second-line treatment may reduce transmission of drug-resistant TB [13], [14]. Transmission models highlighted the importance of considering different elements of the diagnostic pathway (n = 4) including the pre-diagnostic infectious period; others linked operational and transmission models, demonstrating that operational context elements such as patient diagnostic delay, access to care and loss to follow-up can inform setting specific models useful for data-driven policy decisions [15]–[18].

In terms of economic evaluations, models provided comparisons of the cost-effectiveness and impact on TB incidence and mortality of various novel or hypothetical diagnostics and strategies (n = 24) [16], [19]–[41]. Many studies demonstrated the cost-effectiveness of novel diagnostics, often highlighting the settings and implementation strategies and/or algorithms in which those tests were most cost-effective. Among cost-effectiveness models, the most frequently studied diagnostic assays included Xpert MTB/RIF (n = 7) and alternative NAATs (n = 4). Most (n = 16) of the cost-effectiveness models compared new assays to an existing baseline and generated an incremental cost-effectiveness ratio (ICER), of which 69% (n = 11) concluded that novel TB diagnostics were likely to be cost-effective relative to that baseline. Some technologies including NAATs implemented in low TB prevalence settings were judged not to be cost-effective [27],[30], and serology was shown to be more costly and less effective than existing diagnostics such as sputum smear microscopy [28]. Cost-effectiveness studies also highlighted barriers or challenges associated with roll-out of novel diagnostic tests including operational barriers or increased indirect costs, associated with HIV or multi-drug resistance (MDR) care [19], [25]. A transmission study demonstrated that DST for all retreatment cases could be highly cost-effective [40]. Evaluations of hypothetical point of care (POC) tests suggested that a highly specific, low cost POC test would be highly cost-effective, with the greatest impact in settings with poor infrastructure [37].

### Do models include the relevant features?

In each of the three model categories we evaluated relevant features associated with modelling methods, structure and parameters including: outcomes of interest, type of model, sensitivity and uncertainty analyses, costing methods and data sources, how drug susceptibility and HIV status were incorporated in the model along with false negatives and positives.

#### Population impact/transmission models

Sixteen (44%) of the selected papers employed a transmission model to assess the population impact of a novel TB diagnostic [9]–[20], [40], [42]–[44] (Table S1.1 in Data S1). The main outcomes of interest included: TB incidence, TB mortality, incidence of multi-drug resistant TB (MDR-TB) or extensively drug resistant TB (XDR-TB), and transmission rate. All papers focused on implementation in a population with a high TB burden and/or low or middle income setting, with the exception of one theoretical analysis that assessed high, medium and low TB burden settings [15]. Seven of 16 studies (44%) explored methods of TB diagnostic modeling; for example: linking transmission modeling and discrete event simulation (n = 2) [16], [18], including patient–provider interactions (n = 1) [10], assessing implications of periods of pre-diagnostic transmission (n = 1) [15] or modeling details of the diagnostic pathway or diagnostic delay (n = 3) [9], [11], [17]. A dynamic compartmental model was employed in 7/16 (44%) of the transmission studies evaluated [9], [12], [15], [17], [42]–[44]; the remaining studies linked a compartmental model with an operational model (n = 3) [13], [16], [18], a Markov model (n = 1) [10], a state transition model [40], a cost-effectiveness analysis (n = 1) [19], two decision analysis models [11], [40], a cohort model (n = 1) [9] or a Markov model with a cost-effectiveness component (n = 1) [20]. Drug susceptibility and HIV status were each considered in 8/16 (50%) studies and 7/16 studies respectively, 4/16 (25%) considered both in the same model [13], [16], [19], [43].

#### Health system models

Only 5/36 (14%) of included papers explicitly modelled interactions between patients and health care providers or health care facilities [10], [13], [16]–[18]. All models assessed populations in settings with a high TB burden and all were published in the last five years (Table S2.2 in Data S1). Outcomes of interest included TB incidence and prevalence, TB mortality and rates of transmission. One study also assessed costs and number of patients cured [16]. This was the only study from this group to evaluate novel diagnostics (i.e.: LED and Xpert) as opposed to a hypothetical test or a decrease in the delay to diagnosis [16]. All 5 studies employed a transmission model; two employed discrete event simulation [16], [18], one [10] employed a Markov structure and one [13] a queuing structure. Four of 5 studies (80%) [10], [13], [16], [18] explicitly modelled the period of transmission pre-diagnosis (Table S2.3 in Data S1). Two studies [13], [16] included both HIV status and drug susceptibility in the model while a further study considered only HIV status [17].

Two of 5 studies (40%) were calibrated to empirical data [10], [17]: one employing epidemiological characteristics of the TB and HIV epidemic in the modelled country [19] and the other employing study data evaluating patient-provider interactions [17]. One study (20%) considered false positives [16], while 3/5 (60%) explicitly modelled false negatives allowing re-entry into the same diagnostic pathway [10], [16], [17].

Future transmission and health systems models would benefit from careful consideration of the transmission patterns in the pre-diagnostic phase of disease development as well as among individuals receiving false-negative diagnoses. Several factors might contribute to differential transmission during these periods, including the duration of continued infectiousness in each time period, the trajectory of infectiousness over time and changes in contact structure over time. For example, if the majority of the transmission occurs prior to the patient seeking care, improved sensitivity and specificity of a novel diagnostic may not impact upon transmission and therefore TB incidence. Molecular epidemiology studies and emerging technologies including whole genome sequencing coupled with intensive contact tracing studies, may serve to provide improved understanding of these factors in the future, and to ultimately to help better model the impact of novel diagnostics on transmission. Modellers should also carefully consider the trajectory of false negative patients through the diagnostic pathway across different operational contexts. Do false negative diagnoses result (on average) in many months of high-level transmission, just a few weeks of contact only with people who are already exposed, or death with very little on-going transmission? Studies involving intensive follow-up of representative groups of people testing negative for TB in given settings could provide helpful data in understanding how many false negatives ultimately become diagnosed with TB, when diagnosis occurs and what happens to those who are not diagnosed with TB. Data collection in these areas should be encouraged to better inform models seeking to appropriately address these challenging considerations.

#### Cost-effectiveness models

Twenty-four out of the 36 selected papers (67%) included a cost-effectiveness or cost-utility analysis [16], [19]–[41]. Twenty out of 24 studies (83%) were cohort models, including either a decision analysis framework [21]–[23], [25]–[38], [41], or Markov approach [20], [24], [26]; 3/24 (12.5%) were transmission models [16], [19], [40], one study linked a cohort (Markov) and transmission model [20], and one transmission model also utilized a health systems approach [16]. The majority of cost-effectiveness models, 16/24 (67%), took their study population as people with TB symptoms or in the case of DST individuals diagnosed with TB [21]–[23], [25]–[37]; remaining studies assessed HIV populations initiating ART [24], [39], [41], the general population [16], [19], [40], or a prison population with high MDR prevalence [20], [38] (Table S3.1 in Data S1). Most models assessed populations in high TB burden settings, however 5/24 (21%) were set in low TB burden countries [27], [29], [30], [33], [38]. Eight of 24 (33%) studies evaluated Xpert [16], [19]–[21], [24], [32], [34], [36], while 9/24 (37.5%) evaluated an alternative NAAT [22], [27], [29]–[34], [36]. Effectiveness measures in these analyses included health utility measures [quality-adjusted life years (QALYs), 4/24 (17%), or disability-adjusted life years (DALYs), 7/24 (29%)] and cases detected; 16/24 studies (67%) generated an ICER (Table S3.2 in Data S1). Several studies also calculated total program and/or implementation costs. The cost-effectiveness studies were performed from either a health care provider, health system, TB program or TB laboratory perspective. Only one study included patient costs [25], and these were restricted to an approximate estimate of cost of travel to the health facilities. No study reported taking a societal perspective. Less than half (11/24, 46%) employed an empirical costing component in the analysis [16], [20], [22], [25]–[27], [29], [33], [36], [40], [41]. Fifteen of 24 studies (62.5%) included HIV in the model [16], [19]–[21], [23], [24], [26], [28], [32], [34]–[39], [41], and 11/24 (46%) accounted for drug susceptibility status [16], [19], [22], [24], [30], [32]–[34], [36], [38], [40].

There exists a scarcity of models that include the costs incurred by TB patients throughout the diagnostic and treatment process, and no cost-effectiveness studies were conducted from the patient or society perspective. Omission of the patient or societal perspective may underestimate the costs and/or impact associated with earlier or more effective diagnosis. Estimation of patient costs may be important for other models as well; for example, if patient costs of transport and TB diagnosis are catastrophically high, the incremental impact of point-of-care diagnostics (i.e., that do not require multiple visits to healthcare facilities to initiate treatment) may be much greater than if patient costs are low. As TB is a disease of poverty, closer evaluation of the patient-level costs of TB diagnosis may reveal that these costs are a critical barrier to the impact of novel diagnostic tests [45]. Increased effort should be made to collect empirical cost data from the patient and societal perspective in a variety of settings; and/or incorporate existing data into current models.

Moreover, many cost-effectiveness analyses claiming a health systems, health care provider or TB program perspective did not include all relevant costs. Some studies included only test costs and salaries, omitting overhead costs; likewise costs associated with HIV care post-TB diagnosis and MDR-TB treatment post-diagnosis with Xpert or other new diagnostics, were not systematically included across relevant studies. Reviewed studies demonstrated that the inclusion or omission of indirect costs, particularly costs associated with HIV and MDR care, can have important implications for estimated ICERs and model conclusions, and should be carefully considered in cost-effectiveness studies. While often challenging to distinguish the extent to which direct provider costs and higher-level program costs are incremental, omission of these costs may lead to overly optimistic conclusions surrounding the cost-effectiveness of novel interventions. Cost-effectiveness analyses should carefully consider relevant costs and endeavour to include clear explanations of which costs are and are not included and why.

### Is uncertainty properly captured?

Sensitivity analyses were performed in 14/16 transmission studies (87.5%), yet only 6 considered variation in two or more variables at a time [12], [15], [17], [19], [40], [43] (Table S1.3 in Data S1). Half of the papers calibrated their models to existing real data (e.g. incidence trends or point estimates). Among the 16 transmission studies evaluated, 13 (81%) explicitly accounted for the impact of transmission before diagnosis. The impact of individuals with false negative diagnoses (i.e. patients with active TB who were misdiagnosed as TB negative and therefore continued to transmit) was estimated in 6/16 studies (37.5%) [10], [11], [16], [17], [19], [43], of which 5 assumed that these patients would re-enter into the same diagnostic pathway at the same rate as ‘pre-diagnostic’ individuals with active TB. Three health systems studies (60%) performed sensitivity analyses [13], [16], [17], only one of which considered more than one variable at a time [17].

All economic studies performed sensitivity analyses, and 13/24 (54%) assessed sensitivity to more than one variable at a time [19], [20], [22], [23], [26]–[28], [33]–[37], [39], [40] (Table S3.4 in Data S1). Fourteen of 24 (58%) explicitly modelled false positives [16], [19], [22], [24], [26]–[28], [30], [31], [33], [35], [39], [41] (which may have an impact on costs as patients then receive unnecessary treatment and follow-up), 14/24 (58%) studies explicitly modelled false negatives [16], [19], [20], [22]–[24], [26], [28], [30], [31], [34], [36], [39], [41] (which may have an impact on health outcomes as they remain undiagnosed, untreated and potentially infectious), Only three studies allowed false negatives to re-enter the diagnostic pathway, in two cases entering the same diagnostic pathway as those entering for the first time [16], [26] and in the third a second pathway for false negatives was modelled [39].

Sensitivity analyses were frequently limited to one- or two- way analyses. More recent models increasingly include multi-way sensitivity analyses as well; however, thorough investigation and discussion of both uncertainty in model structures and parameter values (both point estimates and associated ranges) remains a largely unmet need. More transparent approaches to uncertainty may help to build understanding and trust in modelling approaches among policy makers.

Most models evaluated do not generate predictions that can be validated against data collected in the future; therefore this systematic review is limited in its ability to assess whether model predictions could be validated in real life. This review is also limited by its ability to review only English language publications.

## Conclusion

As highlighted in this review, models have provided helpful insight both in terms of pragmatic policy implications concerning the cost-effectiveness of novel diagnostics and methodological insights. Models have highlighted the scenarios and algorithms where novel technologies are most cost-effective, demonstrating that while Xpert and other NAATs are highly cost-effective in high TB burden settings, they are not cost-effective in settings with low TB prevalence [19], [20], [27], [30], [36]. Similarly, serology in India was shown to be both more costly and less effective than standard smear microscopy contributing to important policy implications to discourage use of serology at the national and global levels [28]. Models have provided insight on the potential impact of novel diagnostics on transmission of TB over time; implementation of Xpert in five African countries was shown to have the potential to reduce TB incidence and mortality over ten years but increase costs associated with MDR treatment and HIV care [19]. The importance of explicitly modelling the pre-diagnostic infectious period was demonstrated in transmission models that further assessed how different characterizations of this infectious period may have important implications on model results and interpretations. Diagnostic delay was examined in several transmission models, along with the identification of key determinants, such as test sensitivity and the health system's ability to return results to the patient and initiate timely treatment. Finally, models have highlighted the importance of the operational context stressing the need for setting-specific operational or health systems models to guide and support data-driven policy decisions.

The literature on novel TB diagnostic modeling remains limited but is growing rapidly with the on-going development and introduction of novel diagnostic assays and diagnostic systems. We have identified and reviewed a group of models that may help to inform decisions related to implementation of novel TB diagnostics, but further models are needed that include additional epidemiological settings and compare additional diagnostic algorithms. These models would benefit tremendously from epidemiological data on the transmission implications of false-negative diagnosis and the pre-diagnostic period. Improvement and expansion of mathematical models describing transmission, health systems, and cost-effectiveness of novel TB diagnostics will ensure more rational implementation and resource allocation of these tools in order to realize their potential to improve human health in high TB burden settings.

## Supporting Information

Checklist S1 **Prisma Checklist.**
(DOC)

Data S1 **Supplementary tables.** Table S1.1, General overview of population impact/transmission model. Table S1.2, What was modeled (diagnostics and scope of model). Table S1.3, Modeling methods. Table S2.1, Health System models: General overview. Table S2.2, Cost-effectiveness specific considerations. Table S2.3, What was modeled (diagnostics and scope of model). Table S2.4, Modeling methods (including which mixed methods were applied). Table S3.1, Cost-effectiveness models: General overview. Table S3.2, Cost-effectiveness specific considerations. Table S3.3, What was modeled (diagnostics and scope of model). Table S3.4, Modeling methods.
(DOCX)

## References

[1] Boehme CC, Saacks S, O'Brien RJ (2013) The changing landscape of diagnostic services for tuberculosis. Semin Respir Crit Care Med 34: 17–31. 23460003 10.1055/s-0032-1333468

[2] McNerney R, Maeurer M, Abubakar I, Marais B, McHugh TD, et al (2012) Tuberculosis diagnostics and biomarkers: needs, challenges, recent advances, and opportunities. J Infect Dis 205 Suppl 2S147–158. 22496353 10.1093/infdis/jir860

[3] Pai M, Minion J, Steingart K, Ramsay A (2010) New and improved tuberculosis diagnostics: evidence, policy, practice, and impact. Curr Opin Pulm Med 16: 271–284. 20224410 10.1097/MCP.0b013e328338094f

[4] Pai M, O'Brien R (2008) New diagnostics for latent and active tuberculosis: state of the art and future prospects. Semin Respir Crit Care Med 29: 560–568. 18810689 10.1055/s-0028-1085707

[5] Dowdy DW, Cattamanchi A, Steingart KR, Pai M (2011) Is scale-up worth it? Challenges in economic analysis of diagnostic tests for tuberculosis. PLoS Med 8: e1001063. 21814496 10.1371/journal.pmed.1001063 PMC3144197

[6] Bacaer N, Ouifki R, Pretorius C, Wood R, Williams B (2008) Modeling the joint epidemics of TB and HIV in a South African township. J Math Biol 57: 557–593. 18414866 10.1007/s00285-008-0177-z

[7] Garnett GP, Cousens S, Hallett TB, Steketee R, Walker N (2011) Mathematical models in the evaluation of health programmes. Lancet 378: 515–525. 21481448 10.1016/S0140-6736(10)61505-X

[8] Drummond M, Sculpher M, Torrance G, O'Bruen B, Stoddart G (2005) Methods for the economic evaluation of health care programmes. Oxford: Oxford University Press.

[9] Uys PW, Warren RM, van Helden PD (2007) A threshold value for the time delay to TB diagnosis. PLoS One 2: e757. 17712405 10.1371/journal.pone.0000757 PMC1942086

[10] Dye C (2012) The potential impact of new diagnostic tests on tuberculosis epidemics. Indian J Med Res 135: 737–744. 22771607 PMC3401708

[11] Millen SJ, Uys PW, Hargrove J, van Helden PD, Williams BG (2008) The effect of diagnostic delays on the drop-out rate and the total delay to diagnosis of tuberculosis. PLoS One 3: e1933. 18398459 10.1371/journal.pone.0001933 PMC2276686

[12] Legrand J, Sanchez A, Le Pont F, Camacho L, Larouze B (2008) Modeling the impact of tuberculosis control strategies in highly endemic overcrowded prisons. PLoS One 3: e2100. 18461123 10.1371/journal.pone.0002100 PMC2324198

[13] Basu S, Friedland GH, Medlock J, Andrews JR, Shah NS, et al (2009) Averting epidemics of extensively drug-resistant tuberculosis. Proc Natl Acad Sci U S A 106: 7672–7677. 19365076 10.1073/pnas.0812472106 PMC2678614

[14] Uys PW, Warren R, van Helden PD, Murray M, Victor TC (2009) Potential of rapid diagnosis for controlling drug-susceptible and drug-resistant tuberculosis in communities where Mycobacterium tuberculosis infections are highly prevalent. J Clin Microbiol 47: 1484–1490. 19297604 10.1128/JCM.02289-08 PMC2681859

[15] Dowdy DW, Basu S, Andrews JR (2013) Is passive diagnosis enough?: the impact of subclinical disease on diagnostic strategies for tuberculosis. Am J Respir Crit Care Med 187: 543–551. 23262515 10.1164/rccm.201207-1217OC PMC3733406

[16] Langley I, Doulla B, Lin HH, Millington K, Squire B (2012) Modelling the impacts of new diagnostic tools for tuberculosis in developing countries to enhance policy decisions. Health Care Manag Sci 15: 239–253. 22674467 10.1007/s10729-012-9201-3

[17] Lin HH, Dowdy D, Dye C, Murray M, Cohen T (2012) The impact of new tuberculosis diagnostics on transmission: why context matters. Bull World Health Organ 90: 739–747A. 23109741 10.2471/BLT.11.101436 PMC3471051

[18] Lin HH, Langley I, Mwenda R, Doulla B, Egwaga S, et al (2011) A modelling framework to support the selection and implementation of new tuberculosis diagnostic tools. Int J Tuberc Lung Dis 15: 996–1004. 21740663 10.5588/ijtld.11.0062

[19] Menzies NA, Cohen T, Lin HH, Murray M, Salomon JA (2012) Population health impact and cost-effectiveness of tuberculosis diagnosis with Xpert MTB/RIF: a dynamic simulation and economic evaluation. PLoS Med 9: e1001347. 23185139 10.1371/journal.pmed.1001347 PMC3502465

[20] Winetsky DE, Negoescu DM, DeMarchis EH, Almukhamedova O, Dooronbekova A, et al (2012) Screening and rapid molecular diagnosis of tuberculosis in prisons in Russia and Eastern Europe: a cost-effectiveness analysis. PLoS Med 9: e1001348. 23209384 10.1371/journal.pmed.1001348 PMC3507963

[21] Abimbola TO, Marston BJ, Date AA, Blandford JM, Sangrujee N, et al. (2012) Cost-Effectiveness of Tuberculosis Diagnostic Strategies to Reduce Early Mortality Among Persons With Advanced HIV Infection Initiating Antiretroviral Therapy. JAIDS Journal of Acquired Immune Deficiency Syndromes 60: : e1–e7 10.1097/QAI.1090b1013e318246538f. 10.1097/QAI.0b013e318246538f 22240465

[22] Acuna-Villaorduna C, Vassall A, Henostroza G, Seas C, Guerra H, et al (2008) Cost-effectiveness analysis of introduction of rapid, alternative methods to identify multidrug-resistant tuberculosis in middle-income countries. Clin Infect Dis 47: 487–495. 18636955 10.1086/590010

[23] Albert H (2004) Economic analysis of the diagnosis of smear-negative pulmonary tuberculosis in South Africa: incorporation of a new rapid test, FASTPlaqueTB, into the diagnostic algorithm. Int J Tuberc Lung Dis 8: 240–247. 15139454

[24] Andrews JR, Lawn SD, Rusu C, Wood R, Noubary F, et al. (2012) The cost-effectiveness of routine tuberculosis screening with Xpert MTB/RIF prior to initiation of antiretroviral therapy: a model-based analysis. AIDS 26: : 987–995 910.1097/QAD.1090b1013e3283522d3283547. 10.1097/QAD.0b013e3283522d47 PMC3517815 22333751

[25] Bonnet M, Tajahmady A, Hepple P, Ramsay A, Githui W, et al (2010) Added value of bleach sedimentation microscopy for diagnosis of tuberculosis: a cost-effectiveness study. Int J Tuberc Lung Dis 14: 571–577. 20392349

[26] Dowdy DW, Lourenco MC, Cavalcante SC, Saraceni V, King B, et al (2008) Impact and cost-effectiveness of culture for diagnosis of tuberculosis in HIV-infected Brazilian adults. PLoS One 3: e4057. 19129940 10.1371/journal.pone.0004057 PMC2614861

[27] Dowdy DW, Maters A, Parrish N, Beyrer C, Dorman SE (2003) Cost-effectiveness analysis of the gen-probe amplified mycobacterium tuberculosis direct test as used routinely on smear-positive respiratory specimens. Journal of clinical microbiology 41: 948–953. 12624014 10.1128/JCM.41.3.948-953.2003 PMC150318

[28] Dowdy DW, Steingart KR, Pai M (2011) Serological testing versus other strategies for diagnosis of active tuberculosis in India: a cost-effectiveness analysis. PLoS Med 8: e1001074. 21857810 10.1371/journal.pmed.1001074 PMC3153451

[29] Guerra RL, Hooper NM, Baker JF, Alborz R, Armstrong DT, et al (2008) Cost-effectiveness of different strategies for amplified Mycobacterium tuberculosis direct testing for cases of pulmonary tuberculosis. J Clin Microbiol 46: 3811–3812. 18799694 10.1128/JCM.01682-08 PMC2576624

[30] Hughes R, Wonderling D, Li B, Higgins B (2012) The cost effectiveness of Nucleic Acid Amplification Techniques for the diagnosis of tuberculosis. Respiratory medicine 106: 300–307. 22137190 10.1016/j.rmed.2011.10.005

[31] Lim TK, Cherian J, Poh KL, Leong TY (2000) The rapid diagnosis of smear-negative pulmonary tuberculosis: a cost-effectiveness analysis. Respirology 5: 403–409. 11192555

[32] Meyer-Rath G, Schnippel K, Long L, Macleod W, Sanne I, et al (2012) The Impact and Cost of Scaling up GeneXpert MTB/RIF in South Africa. PLoS One 7: e36966. 22693561 10.1371/journal.pone.0036966 PMC3365041

[33] Rajalahti I, Ruokonen EL, Kotomaki T, Sintonen H, Nieminen MM (2004) Economic evaluation of the use of PCR assay in diagnosing pulmonary TB in a low-incidence area. Eur Respir J 23: 446–451. 15065837 10.1183/09031936.04.00009704

[34] Schnippel K, Meyer-Rath G, Long L, Stevens WS, Sanne I, et al (2013) Diagnosing Xpert MTB/RIF negative TB: impact and cost of alternative algorithms for South Africa. S Afr Med J 103: 101–106. 23374320 10.7196/samj.6182

[35] Sun D, Dorman S, Shah M, Manabe YC, Moodley VM, et al (2013) Cost utility of lateral-flow urine lipoarabinomannan for tuberculosis diagnosis in HIV-infected African adults. Int J Tuberc Lung Dis 17: 552–558. 23485389 10.5588/ijtld.12.0627 PMC3918209

[36] Vassall A, van Kampen S, Sohn H, Michael JS, John KR, et al (2011) Rapid diagnosis of tuberculosis with the Xpert MTB/RIF assay in high burden countries: a cost-effectiveness analysis. PLoS Med 8: e1001120. 22087078 10.1371/journal.pmed.1001120 PMC3210757

[37] Dowdy DW, O'Brien MA, Bishai D (2008) Cost-effectiveness of novel diagnostic tools for the diagnosis of tuberculosis. Int J Tuberc Lung Dis 12: 1021–1029. 18713499

[38] Jones TF, Schaffner W (2001) Miniature chest radiograph screening for tuberculosis in jails: a cost-effectiveness analysis. Am J Respir Crit Care Med 164: 77–81. 11435242 10.1164/ajrccm.164.1.2010108

[39] Maheswaran H, Barton P (2012) Intensive case finding and isoniazid preventative therapy in HIV infected individuals in Africa: economic model and value of information analysis. PLoS One 7: e30457. 22291958 10.1371/journal.pone.0030457 PMC3264596

[40] Resch SC, Salomon JA, Murray M, Weinstein MC (2006) Cost-effectiveness of treating multidrug-resistant tuberculosis. PLoS Med 3: e241. 16796403 10.1371/journal.pmed.0030241 PMC1483913

[41] Samandari T, Bishai D, Luteijn M, Mosimaneotsile B, Motsamai O, et al (2011) Costs and consequences of additional chest x-ray in a tuberculosis prevention program in Botswana. Am J Respir Crit Care Med 183: 1103–1111. 21148723 10.1164/rccm.201004-0620OC PMC3159079

[42] Abu-Raddad LJ, Sabatelli L, Achterberg JT, Sugimoto JD, Longini IM Jr, et al (2009) Epidemiological benefits of more-effective tuberculosis vaccines, drugs, and diagnostics. Proc Natl Acad Sci U S A 106: 13980–13985. 19666590 10.1073/pnas.0901720106 PMC2720405

[43] Dowdy DW, Chaisson RE, Maartens G, Corbett EL, Dorman SE (2008) Impact of enhanced tuberculosis diagnosis in South Africa: a mathematical model of expanded culture and drug susceptibility testing. Proc Natl Acad Sci U S A 105: 11293–11298. 18695217 10.1073/pnas.0800965105 PMC2516234

[44] Dowdy DW, Chaisson RE, Moulton LH, Dorman SE (2006) The potential impact of enhanced diagnostic techniques for tuberculosis driven by HIV: a mathematical model. AIDS 20: 751–762. 16514306 10.1097/01.aids.0000216376.07185.cc

[45] Barter DM, Agboola SO, Murray MB, Barnighausen T (2012) Tuberculosis and poverty: the contribution of patient costs in sub-Saharan Africa–a systematic review. BMC Public Health 12: 980. 23150901 10.1186/1471-2458-12-980 PMC3570447

