# Supplementary material for: Modeling of Novel Diagnostic Strategies for Active Tuberculosis – A Systematic Review: Current Practices and Recommendations
Source: PLoS One. 2014 Oct 23;9(10):e110558. doi: 10.1371/journal.pone.0110558 (PMC4207742; doi:10.1371/journal.pone.0110558)
Supplement: Data S1 — Supplementary tables. Table S1.1, General overview of population impact/transmission model. Table S1.2, What was modeled (diagnostics and scope of model). Table S1.3, Modeling methods. Table S2.1, Health System models: General overview. Table S2.2, Cost-effectiveness specific considerations. Table S2.3, What was modeled (diagnostics and scope of model). Table S2.4, Modeling methods (including which mixed methods were applied). Table S3.1, Cost-effectiveness models: General overview. Table S3.2, Cost-effectiveness specific considerations. Table S3.3, What was modeled (diagnostics and scope of model). Table S3.4, Modeling methods. (DOCX) [file pone.0110558.s002.docx]

## Supplementary material

**Detailed search string:** (tuberculosis OR TB) AND ((mathem* AND (model OR models)) OR (mathem* modell*) OR (mathem* modeling) OR (modeling OR modelling) OR "Population Dynamics"[MeSH Terms] OR "Population Dynamics" OR "System Dynamics" OR "Computer Simulation" OR "Computer Simulation"[MeSH Terms] OR (epidemiological AND “model”))

**Abbreviations used in following tables:**

Inc: incidence

Prev: prevalence

CE: cost-effectiveness

MDR: multi-drug resistance

DST: drug susceptibility testing

XDR: extensively drug resistant

SSpos/neg: sputum smear positive or negative

HYPO: hypothetical test

Symp: symptom screening

Dx: diagnosis

Rx: treatment

pre-diag infect: period of infectiousness pre-diagnosis

drug susc: drug susceptibility

ICER: incremental cost-effectiveness ratio

DALY: disability adjusted life year

**Table S1.1**: General overview of population impact/transmission model (n=16)

| **Reference** | **Primary research question** | **Population** | **Setting** | **Baseline diagnostic pathway** | **Main comparison** | **Outcome** | **Time horizon** | **Conclusion** |
| --- | --- | --- | --- | --- | --- | --- | --- | --- |
| **Abu-Raddad PNAS 2009** | Potential impact of novel vaccine, drugs and diagnostics | General pop'n | Southeast Asia (not China) | Assumed standard DOTS (Sputum smear & Xray for smear negative) | LED, NAAT, dipstick test | TB inc, mortality | 35 yrs | NAAT prevents equal number of deaths as LED, but prevents twice as many cases |
| **Basu 2009 PNAS** | Evaluating transmission dynamics of XDR-TB in South Africa | General pop'n | KwaZulu-Natal (South Africa) | Clinical Dx of DR | Rapid DST for all new TB cases (turnover reduced from 6 wks to <1 wk) | Transmission, mortality | 5 yrs | Early community based DST could help reduce ongoing transmission of DR TB |
| **Dowdy 2006 AIDS** | Impact of improved diagnostics on TB incidence in high HIV prevalence settings | General pop'n | High HIV prevalence | Current standard Diagnostic: sens: 80% SSpos, 25% SSneg | 1) Rapid molecular testing  2) culture | Mortality & TB Inc/Prev | 16-32 yrs | Improved diagnositics may have substantial impact on TB morbidity and mortality in HIV-endemic regions |
| **Dowdy 2008 PNAS** | Impact of enhanced TB diagnostics on the TB epidemic in South Africa | General pop'n | South Africa | Culture without DST performed in 5% of new suspects and with DST in 37% suspects with previous treatment | Culture in all suspects, DST in 37% of new suspects, 85% of retreatment suspects & hypothetical test | Mortality, MDR/XDR TB incidence | 10 yrs | Rapid expansion of culture and DST reduces overall mortality (17%) and MDR mortality (47%), but does not prevent XDR incidence |
| **Dowdy 2013 AJRCCM** | Estimate pop'n level impact of TB case-finding strategies in presence of subclinical prediagnostic disease | General pop'n | Low, medium and high burden | *Assumed standard DOTS (Sputum smear & Xray for smear negative)* | Increased sensitivity during the clinical phase | TB inc | 10 yrs | Pre-diagnostic infectious period important to include when evaluating diagnostic and case finding strategies |
| **Dye 2012 IJMR** | Explore potential impact of new TB diagnostic tests on TB epidemic | General pop'n | India | N/A | Diagnostic pathway that halves diagnostic delay | TB inc | 40 yrs | New diagnostic test will most reduce diagnostic delay when applied by all providers (public and private) |
| **Langley 2012 HCMS** | Explore how discrete event simulation can inform implementation decisions around novel diagnostics | General pop'n | Tanzania | Sputum smear & DST in reference lab | 1) full implementation of NAAT (Xpert) 2) LED optimized microscopy | Costs, patients cured | Lifetime, 10 yrs | Linked operational and transmission model highly useful to inform policy decisions on TB diagnostics |
| **Legrand 2008 PLosONE** | Explore impact of TB control strategies (including screening with MMR) on prevalence of active TB | Prison inmate population | Rio de Janeiro, Brazil | No active surveillance | Mass annual X-Ray, entry X-Ray, symptom screen at entry, DOTS | Active TB prevalence | 10 yrs | DOTS strategy with annual mass X-Ray screening would obtain a rapid and sustained decline in prevalence of active TB |
| **Lin 2011 IJTLD** | Potential of integrating operational and dynamic transmission model | General pop'n | Low- and middle- income countries | Sputum smear | Hypothetical faster and more sensitive test | TB inc | 10 yrs | Linked operational and transmission model useful to inform impact of alternative diagnostic pathways |
| **Lin 2012 BullWHO** | Estimate impact of new diagnostic tool in detailed model of diagnostic pathway | General pop'n | Tanzania | Sputum smear & Xray for smear negative | Hypothetical first line test 100/70% sensitivity for SSpos/SSneg TB | TB inc, prev | 10 yrs | Models of diagnostic impact should include operational context |
| **Menzies 2012 PLosMed** | Population impact and CE of Xpert for TB diagnosis | General pop'n | Botswana, Lesotho, Namibia, South Africa, Swaziland | Sputum smear, culture if - on smear & strong suspicion of TB or history of TB treatment | Xpert as first line test | TB inc, prev | 10 & 20 yrs | Introduction of Xpert would lower incidence, prevalence and mortality within 10 yrs, but will increase costs for HIV care and MDR treatment |
| **Millen 2008 PLosONE** | Impact of test sensitivity on diagnostic delay and drop out | TB Cases | Parameters based on South Africa (Western Cape) | Sputum smear & Xray for smear negative, culture centralised | One stop test with 60% sensitivity | Diagnostic delay | Diagnostic pathway | Test sensitivity is key determinant of diagnostic delay |
| **Resch 2006 PLosMed** | Assess CE of diagnosing and treating MDR TB in LMIC | General pop’n | Peru (LMIC) | No DST, multiple courses of first line Tx | DST for previously treated pts and individualized treatment plans | $/QALY saved | 30 yrs | DST for all retreatment cases is highly cost-effective |
| **Uys 2007 PlosONE** | Impact of diagnostic delay on transmission | General pop'n | South Africa (Western Cape) | N/A | Reduction in diagnostic delay that decreases rate of infection of personal contacts by 20% | Transmission | ~15 wks | Average time to diagnosis needs to be below a threshold, otherwise an epidemic will escalate |
| **Uys 2009 JCM** | Impact of delayed diagnosis of DR in TB patients | General pop'n | Western Cape (South Africa) | Culture for DST (turnover of 40 days) | MTBDRplus (2 day turnover) | TB inc  (DR TB) | 20 yrs | Current strategies have long delays and will not halt the spread of MDR TB, rapid diagnosis of drug reisstance in the community is needed |
| **Winetsky 2012 PLosMed** | Evaluate CE of Xpert and other Dx strategies in prisons in Russia and Eastern Europe | Prison pop’n with high MDR prevalence | Tajikistan, Russia, Latvia | No screening | Annual mass screen with Xpert or MMR | TB and MDR prev, costs | 10 yrs, lifetime | Annual screening with Xpert is more effective than MMR and is cost-effective |

**Table S1.2**: What was modeled (diagnostics and scope of model)

| **Reference** | **Novel diagnostic test** | | **Assessed modeling methods?** | | **Diagnostic tools explicitly modeled** | | | | | | | **Stage of technology** | **Health system scope** |
| --- | --- | --- | --- | --- | --- | --- | --- | --- | --- | --- | --- | --- | --- |
|  |  |  |  |  | **Symp** | **Sputum Smear** | **Xray** | **Xpert** | **Other NAAT** | **Culture** | **Other** |  |  |
| **Abu-Raddad PNAS 2009** | Y | HYPO*: LED, NAAT, Dipstick | N | N/A | N | Y (LED) | N | N | Non-specific | N | HYPO: dipstick | Scale-up/ Product profile | Dx Rx |
| **Basu 2009 PNAS** | Y | HYPO: Rapid DST | N | N/A | Y | Y | Y | N | N | N | HYPO: Rapid POC test for XDR | Product profile | Dx Rx |
| **Dowdy 2006 AIDS** | Y | Rapid molecular testing, culture | N | N/A | N | Y | N | N | Non-specific | Y | N/A | Product profile | Other services (HIV) |
| **Dowdy 2008 PNAS** | Y | Expanded culture and DST | N | N/A | N | Y | Y | N | N | Y | HYPO: 100% sensitivity, immediate result, 1m drug resistance result | Scale up/ Product profile | Dx Rx |
| **Dowdy 2013 AJRCCM** | Y | HYPO: 3 Dx tests | Y | Pre-Dx transmission | N | N | N | N | N | N | HYPO: 20% increase in in sens (similar to Xpert) | Product profile | Dx Rx |
| **Dye 2012 IJMR** | Y | HYPO | Y | Include interactions between patient and provider | N | N | N | N | N | N | HYPO: improved test | Product profile | Dx Rx |
| **Langley 2012 HCMS** | Y | Xpert | Y | Link operational and transmission model | Y | Y  (ZN & LED) | Y | Y | N | Solid | N/A | Existing test | Dx Rx |
| **Legrand 2008 PLosONE** | Y | Mass X-Ray screening | N | N/A | N | Y | Y | N | N | N | N | Existing test | Dx Rx |
| **Lin 2011 IJTLD** | N | N/A | Y | Link operational and transmission model | N | N | N | N | N | N | HYPO: 1 sample 1 day test | Product profile | Dx Rx |
| **Lin 2012 BullWHO** | Y | HYPO | Y | More detail of diagnostic pathway | N | Y | Y | N | N | N | HYPO: 100% sens for smear + 70% smear | Product profile | Dx Rx |
| **Menzies 2012 PlosMed** | Y | Xpert | N | N/A | Y | Y | Y | Y | N | Y | N/A | Existing test / Scale-up | other services (HIV) |
| **Millen 2008 PLosONE** | N | N/A | Y | Diagnostic delay | N | Y | Y | N | N | Solid | N/A | Product profile | Dx |
| **Resch 2006 PLosMed** | Y | DST | N | N/A | N | N | N | N | N | DST | N | Existing test | Dx Rx |
| **Uys 2007 PlosONE** | N | N/A | Y | Diagnostic delay | N | N | N | N | N | N | N/A | Product profile | Dx |
| **Uys 2009 JCM** | Y | MTBDRplus | N | N/A | N | N | N | N | MTBDRplus | N | N/A | Existing test | Dx Rx |
| **Winetsky 2012 PloS Med** | Y | Xpert, mass miniature radiography (MMR) | N | N/A | Y | N | Y (MMR) | Y | N | Liquid & Solid | N/A | Existing test | Dx Rx |

**Table S1.3:** Modeling methods

| **Reference** | **Model type(s)** | **Health System** | **Data fit** | **Sensitivity** | **Pre-diag inf** | **False +** | **False -** | **Repeat entry** | | **Drug Susc** | **HIV** | **Previous Treatment** |
| --- | --- | --- | --- | --- | --- | --- | --- | --- | --- | --- | --- | --- |
| **Abu-Raddad PNAS 2009** | Transmission | N | Y | one-way | Y | N | N | N | N/A | Y | N | N |
| **Basu 2009 PNAS** | Transmission & queuing | Y | N | unclear | Y | N | N | N | N/A | Y | Y | Y |
| **Dowdy 2006 AIDS** | Transmission | N | N | one-way | Y | N | N | N | N/A | N | Y | N |
| **Dowdy 2008 PNAS** | Transmission | N | Y | multi | Y | N | Y | Y | Identical | Y | Y | Y |
| **Dowdy 2013 AJRCCM** | Transmission | N | N | multi | Y | N | N | N | N/A | N | N | N |
| **Dye 2012 IJMR** | Transmission & markov | Y | Y | None | Y | N | Y | Y | Identical | N | N | N |
| **Langley 2012 HCMS** | Transmission & discrete event simulation | Y | N | one-way | Y | Y | Y | Y | Identical | Y | Y | Y |
| **Legrand 2008 PLosONE** | Transmission | N | Y | Multi | N | N | N | N | N/A | N | N | Y |
| **Lin 2011 IJTLD** | Transmission & discrete event simulation | Y | N | None | Y | N | N | N | N/A | N | N | N |
| **Lin 2012 BullWHO** | Transmission | Y | Y | multi | Y | N | Y | Y | Identical | N | Y | Y |
| **Menzies 2012 PlosMed** | Transmission and CE | N | Y | multi | Y | Y | Y | N | N/A | Y | Y | Y |
| **Millen 2008 PLosONE** | Decision analytic | N/A | N/A | one-way | N | N | Y | Y | Identical | N | Y | N |
| **Resch 2006 PLosMed** | Transmission | N | Y | Two-way | N | N | N | N | N/A | Y | N | Y |
| **Uys 2007 PlosONE** | Transmission (cohort model) | N | N | one-way | Y | N | N | N | N/A | N | N | N |
| **Uys 2009 JCM** | Transmission | N | Y | one-way | Y | N | N | N | N/A | Y | N | N |
| **Winetsky 2012 PLosMed** | Transmission & markov with CE | N | Y | one-way | Y | Y | N | N | N/A | Y | N | Y |

**Table S2.1:** Health System models: General overview (n=5)

| **Ref Reference** | **Primary research question** | **Population** | **Setting** | **Baseline diagnostic pathway** | **Main comparison** | **Outcome** | **Time horizon** | **Conclusion** |
| --- | --- | --- | --- | --- | --- | --- | --- | --- |
| **Basu 2009 PNAS** | Evaluating transmission dynamics of XDR-TB in South Africa | General pop'n | South Africa (KwaZulu-Natal) | Clinical diagnosis of drug resistance | Rapid DST for all new TB cases (turnover reduced from 6 wks to <1 wk) | Transmission, mortality | 5 yrs | Early community based DST could help reduce ongoing transmission of DR TB |
| **Dye 2012 IJMR** | Explore potential impact of new TB diagnostic tests on TB epidemic | General pop'n | India | N/A | Dx pathway that halves diagnostic delay | TB inc | 40 yrs | New diagnostic test will most reduce diagnostic delay when applied by all providers (public and private) |
| **Langley 2012 HCMS** | Explore how discrete event simulation can inform implementation decisions around novel diagnosis | General pop'n | Tanzania | Sputum smear & DST in reference lab | 1) full implementation of NAAT (Xpert) 2) LED optimized microscopy | Costs, patients cured | Lifetime, 10 yrs | Linked operational and transmission model highly useful to inform policy decisions on TB diagnostics |
| **Lin 2011 IJTLD** | Potential of integrating operational and dynamic transmission model | General pop'n | Low- and middle- income | Sputum smear | Hypothetical faster and more sensitive test | TB inc | 10 yrs | Linked operational and transmission model useful to inform impact of alternative diagnostic pathways |
| **Lin 2012 BullWHO** | Estimate impact of new diagnostic tool in detailed model of diagnostic pathway | General pop'n | Tanzania | Sputum smear & Xray for smear negative | Hypothetical first line test 100/70% sensitivity for SSpos/SSneg TB | TB inc, prev | 10 yrs | Models of diagnostic impact should include operational context |

**Table S2.2:** Cost-effectiveness specific considerations

| **Reference** | **CE included** | | | **Costing perspective** | | | **Costing Source** | **Costing Scope** |
| --- | --- | --- | --- | --- | --- | --- | --- | --- |
|  | **Done** | **CE measure** | **ICER** | **Health system vs TB program** | **Patient/family** | **Society** |  |  |
| **Basu 2009 PNAS** | N | N/A | N/A | N/A | N/A | N/A | N/A | N/A |
| **Dye 2012 IJMR** | N | N/A | N/A | N/A | N/A | N/A | N/A | N/A |
| **Langley 2012 HCMS** | Y | $/DALY | Y | Health system | N | N | primarily empirical | full site |
| **Lin 2011 IJTLD** | N | N/A | N/A | N/A | N/A | N/A | N/A | N/A |
| **Lin 2012 BullWHO** | N | N/A | N/A | N/A | N/A | N/A | N/A | N/A |

**Table S2.3:** What was modeled (diagnostics and scope of model)

| **Reference** | **Novel diagnostic** | | **Assessed modeling methods?** | | **Diagnostic tools explicitly modeled** | | | | | | | **Stage of technology** | **Health system scope** |
| --- | --- | --- | --- | --- | --- | --- | --- | --- | --- | --- | --- | --- | --- |
|  |  |  |  |  | **Symp** | **Sputum Smear** | **Xray** | **Xpert** | **Other NAAT** | **Culture** | **Other** |  |  |
| **Basu 2009 PNAS** | Y | HYPO: Rapid DST | Y | Transmission & queuing | Y | Y | Y | N | N | N | HYPO: Rapid POC test for XDR | Product profile | Dx Rx |
| **Dye 2012 IJMR** | Y | HYPO | Y | Transmission & markov | N | N | N | N | N | N | HYPO: improved test | Product profile | Dx Rx |
| **Langley 2012 HCMS** | Y | Xpert | Y | Transmission & discrete event simulation | Y | Y  (ZN & LED) | Y | Y | N | Solid | N/A | Existing test | Dx Rx |
| **Lin 2011 IJTLD** | N | N/A | Y | Transmission & discrete event simulation | N | N | N | N | N | N | HYPO: 1 sample 1 day test | Product profile | Dx Rx |
| **Lin 2012 BullWHO** | Y | HYPO | N | N/A | N | Y | Y | N | N | N | HYPO: 100% sens for smear + 70% smear | Product profile | Dx Rx |

**Table S2.4:** Modeling methods (including which mixed methods were applied)

| **Reference** | **Model type(s)** | **Health System** | **Data fit** | **Sensitivity** | | **Pre-diag**  **infect** | **False +** | **False -** | **Repeat entry** | | **Drug**  **Susc** | **HIV** | **Previous Treatment** |
| --- | --- | --- | --- | --- | --- | --- | --- | --- | --- | --- | --- | --- | --- |
| **Basu 2009 PNAS** | Transmission & queuing | Y | N | unclear | Y | | N | N | N | N/A | Y | Y | Y |
| **Dye 2012 IJMR** | Transmission & markov | Y | Y | None | Y | | N | Y | Y | Identical | N | N | N |
| **Langley 2012 HCMS** | Transmission & discrete event simulation | Y | N | one-way | Y | | Y | Y | Y | Identical | Y | Y | Y |
| **Lin 2011 IJTLD** | Transmission & discrete event simulation | Y | N | None | N | | N | N | N | N/A | N | N | N |
| **Lin 2012 BullWHO** | Transmission | Y | Y | multi | Y | | N | Y | Y | Identical | N | Y | Y |

**Table S3.1:** Cost-effectiveness models: General overview (n=24)

| **Reference** | **Primary research question** | | **Population** | | **Setting** | | **Baseline diagnostic pathway** | **Main comparison** | **Outcome** | | **Time**  **horizon** | **Conclusion** |
| --- | --- | --- | --- | --- | --- | --- | --- | --- | --- | --- | --- | --- |
| **Abimbola 2012 AIDS** | CE of culture or Xpert to reduce early mortality in individuals with advanced HIV initiating ART | | HIV positive individuals initiating ART with TB symptoms | | Sub-Saharan Africa | | Sputum smear microscopy, Xray if SSneg | Xpert as first line test | Mortality, costs | | 6 months | Culture or Xpert CE at reducing early mortality during first 6 months of ART compared with sputum smear & Xray |
| **Acuna CID 2008** | CE of DST including rapid (FASTPlaque) or conventional methods | | TB Cases (Sspos PTB) | | Peru (middle income) | | No DST, MDR Rx based on failure with first line Rx | FASTPlaque-Response, INNO-LiPA, direct LJ, MIT assay indirect LJ | Mortality, costs | | Lifetime | All alternative DST methods are CE, solid culture is most cost-effective |
| **Albert 2004 IJTLD** | CE of incorporating FASTPlaqueTB into Dx algorithm for SS- PTB in South Africa | | TB Suspects (Ssneg) | | Cape Town (South Africa) | | Negative sputum smear (2x), Xray + culture if Xray abnormal | FASTPlaque integrated with Dx pathway | Costs, Cases Dx | | Diagnostic pathway | FASTPlaqueTB improves case-detection and is cheaper to implement than current NTP algorithm |
| **Andrews 2012 AIDS** | CE of Xpert TB screening at ART initiation | | HIV positive individuals initiating ART | | South Africa | | No TB screening | One or two sample Xpert | Survival | | Lifetime | All strategies increased life expectancy, at 5100 USD per life year saved with 2 sample Xpert and 2800 USD for sputum smear |
| **Bonnet 2010 IJTLD** | CE of sputum smear methods that apply bleach sedimentation | | TB Suspects | | Kenya (urban health clinic) | | Sputum smear | Bleach sedimentation on sputum samples | Costs, case detection rate | | Diagnostic pathway | Bleach sedimentation could be CE, but operational barriers complicate roll-out |
| **Dowdy 2003 JCM** | CE of GenProbe for rapid exclusion of *Mtb* in smear positive specimens | | TB Suspects (Sspos) | | Baltimore (USA) | | Sputum smear | GenProbe to exclude *Mtb* in positive smears and avoid isolation | Costs | | Diagnostic pathway | Gen-Probe not CE for most hospitals in high-income setting |
| **Dowdy 2008 IJTLD** | CE of hypothetical new POC test for TB | | TB Suspects (for PTB) | | South Africa, Brazil, Kenya | | No microscopy | Combination of sputum smear, culture, new test with sens = 50-90% and spec = 90-100% | Costs, infections prevented | | Lifetime | Novel Dx can be highly CE. Impact highest from highly specific, low-cost tests in setting with poor infrastructure |
| **Dowdy 2008 Plos ONE** | CE of TB culture for HIV positive patients | | TB Suspects (HIV positive) | | Rio de Janeiro (Brazil) | | Sputum smear | Sputum smear & culture | Mortality, costs | | Lifetime | TB culture is potentially cost-effective diagnostic tool for diagnosis in HIV positive individuals |
| **Dowdy 2011 PlosMed** | CE of TB serology tests in India | | TB Suspects | | India | | No microscopy | Sputum smear vs Anda tb (serology Elisa) | Mortality, costs | | Lifetime | Sputum smear is more cost-effective than serological tests |
| **Guerra 2008 JCM** | CE of specimen dilution algorithms for amplified MTD testing of respiratory specimens | | TB suspects (with smear result) | | Baltimore (USA) | | Conventional undiluted MTD | Various algorithms on diluting sputum samples before MTD | Costs | | Diagnostic pathway | Most CE strategy was dilution for Sspos but not Ssneg specimens prior to MTD testing |
| **Hughes 2012 RespMed** | CE of NAAT based strategies for TB diagnosis | | TB suspects | | UK | | Sputum smear and culture | NAAT as first line or as part of algorithm with Sputum smear, culture, NAAT | Costs | | Lifetime | NAAT based diagnosis not CE below pre-test TB prevalence of 46% |
| **Jones 2001 AJRCCM** | Cost-effectiveness of MMR screening compared with symptom screen or TST | | Prison Inmates | | USA | | No screening for active TB | MMR screening | $/case identified | | End of Rx | Screening with MMR costs less per case identified compared with TST and symptom screening |
| **Langley 2012 HCMS** | Explore how discrete event simulation can inform implementation decisions around novel Dx | | General pop'n | | Tanzania | | Sputum smear & DST in reference lab | 1) full implementation of NAAT (Xpert) 2) LED optimized microscopy | Costs, patients cured | | Lifetime, 10 yrs | Linked operational and transmission model highly useful to inform policy decisions on TB diagnostics |
| **Lim 2000 Resp** | CE of empirical versus lab test (including NAAT) driven diagnosis of smear negative TB | | TB suspects (SSneg PTB) | | Singapore | | Clinical signs only | Amplicor and NAAT for BAL | Costs, survival | | Lifetime | Compared with clinical signs only, additional testing (Amplicor) provides little improvement in life expectancy. |
| **Maheswaran 2011 PLosONE** | Assess CE of active screening strategies for TB in PLWH pre-IPT | | HIV positive population | | sub-Saharan Africa | | Screening for chronic cough | Screening with symptom, sputum and Xray | $/QALY saved | | 2 yrs | Screening those with symptoms with sputum smear is the least costly and most cost-effective option. Addition of CXR is not beneficial. |
| **Menzies 2012 PlosMed** | Population impact and CE of Xpert for TB diagnosis | | General pop'n | | Botswana, Lesotho, Namibia, South Africa, Swaziland | | Sputum smear, culture if - on smear & strong suspicion of TB or hx of TB Rx | Xpert as first line test | TB inc, prev | | 10 & 20 yrs | Introduction of Xpert would lower incidence, prevalence and mortality within 10 yrs, but will increase costs for HIV care and MDR Rx. |
| **Meyer-Rath 2012 PLosONE** | Cost and impact of national rollout of Xpert in South Africa | | TB suspects | | South Africa | | Sputum smear, Xray, centralised culture facility | Xpert as first line test | Costs, cases diagnosed, | | Diagnostic pathway | In Xpert algorithm, cost per diagnosis increased with 55%, diagnosed 30-37% more cases |
| **Rajalahti 2004 ERJ** | Compare standard sputum smear+culture with PCR included strategy | | TB Suspects | | Finland | | Sputum smear & culture | Amplicor standard immediately after first smear and culture | Costs | | End of Rx | Routine PCR not cost saving in low prevalence setting |
| **Resch 2006 PLosMed** | Assess CE of diagnosing and treating MDR TB in LMIC | General pop’n | | Peru (LMIC) | | No DST, multiple courses of first line Tx | | DST for previously treated pts and individualized treatment plans | | $/QALY saved | 30 yrs | DST for all retreatment cases is highly cost-effective |
| **Samandari 2011 AJRCCM** | Assess CE of adding CXR to symptom screening pre-IPT in PLHIV | Persons living with HIV (PLWH) | | Botswana | | Symptoms screen only | | Symptom screen and CXR | | $/case averted | 3 yrs | Symptom screening alone prevents more TB cases and is less costly compared with symptom screen and CXR |
| **Schnippel 2013 SAMJ** | Cost and impact of second Xpert for HIV positive TB supects negative on first Xpert | | TB suspects (HIVpos, initial Xpert negative) | | South Africa | | Culture when negative on initial Xpert | Replace culture with second Xpert | Costs, cases diagnosed | | End of Rx | Second Xpert could improve outcomes and generate cost savings |
| **Sun 2013 IJTLD** | CE of adding LAM urine test to Dx algorithm for individuals with advanced HIV | | TB suspects (HIVpos, CD4<100, 1 TB symptom) | | South Africa & Uganda | | Standard Dx pathway, 35/99.8% sens/spec | Urine LAM added | Costs, cases diagnosed | | Lifetime | Adding urine LAM generated additional Dx and is likely to be CE |
| **Vassall 2011 PLosMed** | CE of Xpert in high burden settings | | TB Suspects | | India, South Africa, Uganda | | Sputum Smear (clinical diagnosis for SSneg) and culture based DST for retreatment cases | 1) Xpert in addition to smear  2) Xpert replaces smear | Costs | | Lifetime | Xpert as a first line test is CE for the diagnosis of TB in low- and middle-income settings, compared smear and clinical signs |
| **Winetsky 2012 PLosMed** | Evaluate CE of Xpert and other Dx strategies in prison populations in Russia and Eastern Europe | | Prison pop’n with high MDR prevalence | | Tajikistan, Russia, Latvia | | No screening | Annual mass screen with Xpert or MMR | TB and MDR prev, costs | | 10 yrs, lifetime | Annual screening with Xpert is more effective than MMR and is cost-effective |
|  |  | |  | |  | |  |  |  | |  |  |

**Table S3.2:** Cost-effectiveness specific considerations

| **Reference** | **Model method** | **CE**  **measure** | **ICER** | **Costing perspective** | | | **Costing Source** | **Costing Scope** |
| --- | --- | --- | --- | --- | --- | --- | --- | --- |
|  |  |  |  | **Health system vs TB program** | **Patient/family** | **Society** |  |  |
| **Abimbola 2012 AIDS** | Decision | $/death averted | Y | Health system | N | N | primarily non-empirical | full site (ART) |
| **Acuna CID 2008** | Decision | $/DALY | N | Health system | N | N | primarily empirical | full site |
| **Albert 2004 IJTLD** | Decision | $/SSneg suspect tested | N | Health system | N | N | primarily non-empirical | full site |
| **Andrews 2012 AIDS** | Markov | $/YLS | Y | Health system | N | N | combo | full site (HIV costs) |
| **Bonnet 2010 IJTLD** | Decision | $/case detected | Y | Health system | Y (transport costs) | N | primarily empirical | full site |
| **Dowdy 2003 JCM** | Decision | $/early TB exclusion | N | Health system | N | N | primarily empirical | full site |
| **Dowdy 2008 IJTLD** | Decision | $/DALY | Y | TB program (costs for hospitalizations or physician visits not included) | N | N | primarily non-empirical | partial site |
| **Dowdy 2008 Plos ONE** | Decision & Markov | $/DALY | Y | TB program | N | N | primarily empirical | full site |
| **Dowdy 2011 PLosMed** | Decision | $/DALY | Y | TB program (public and private) | N | N | primarily non-empirical | partial site (but do include some capital costs) |
| **Guerra 2008 JCM** | Decision | $/correct PTB Dx | N | TB lab perspective | N | N | primarily empirical | partial site |
| **Hughes 2012 RespMed** | Decision | $/QALY | Y | Health system | N | N | primarily non-empirical | partial site |
| **Jones 2001 AJRCCM** | Decision | $/case detected | Y | Health system | N | N | Primarily non-empirical | partial site |
| **Langley 2012 HCMS** | Transmission & discrete event simulation | $/DALY | Y | Health system | N | N | primarily empirical | full site |
| **Lim 2000 Resp** | Decision | $/yr added life expectancy | N | Health system | N | N | combo | partial site |
| **Maheswaran 2011 PLosONE** | Individual sampling model | $/QALY | Y | TB program | N | N | Primarily non-empirical | partial site |
| **Menzies 2012 PlosMed** | Transmission and CE | $/DALY | Y | Health system | N | N | primarily non-empirical | above service level (HIV) |
| **Meyer-Rath 2012 PLosONE** | Decision | $/case treated & $/suspect | Y | Health system | N | N | combo | full site |
| **Rajalahti 2004 ERJ** | Decision | $/pt tested | Y | Health system | N | N | primarily empirical | partial site/full site but doesn't specify salaries, overhead, etc. |
| **Resch 2006 PLosMed** | Transmission | $/QALY saved | Y | Health system | N | N | primarily empirical | Partial site |
| **Samandari 2011 AJRCCM** | Decision | $/case averted | Y | Health system | N | N | primarily empirical | Partial site |
| **Schnippel 2013 SAMJ** | Decision | $/TB case initiated on Rx | N | Health system | N | N | primarily non-empirical (uses WHO CHOICE) | partial site |
| **Sun 2013 IJTLD** | Decision | $/DALY | Y | TB program | N | N | primarily non-empirical | partial site |
| **Vassall 2011 PLosMed** | Decision | $/DALY | N | Health system | N | N | primarily empirical | full site |
| **Winetsky 2012 PLosMed** | Transmission & markov with CE | $/QALY | Y | Health system | N | N | primarily empirical | full site |

**Table S3.3:** What was modeled (diagnostics and scope of model)

| **Reference** | **Novel diagnostic** | | **Assessed modeling methods?** | | | **Diagnostic tools explicitly modeled** | | | | | | | **Stage of technology** | **Health system scope** |
| --- | --- | --- | --- | --- | --- | --- | --- | --- | --- | --- | --- | --- | --- | --- |
|  |  |  |  |  |  | **Symp** | **Sputum Smear** | **Xray** | **Xpert** | **Other NAAT** | **Culture** | **Other** |  |  |
| **Abimbola 2012 AIDS** | Y | Xpert | | N | N/A | N | Y | Y | Y | N | Liquid | N/A | Existing test | Other services (HIV) |
| **Acuna CID 2008** | Y | FASTPlaque-Response, INNO-LiPA, direct LJ, MIT assay indirect LJ | | N | N/A | N | N | N | N | LPA | Solid | FASTPlaque-Response, INNO-LiPA, MTT(colorimetric) | Existing test | Dx Rx |
| **Albert 2004 IJTLD** | Y | FASTPlaqueTB | | N | N/A | N | Y | Y | N | N | Liquid | FASTPlaqueTB | Existing test | Dx |
| **Andrews 2012 AIDS** | Y | Xpert | | N | N/A | Y | Y | Y | Y | N | Liquid | HYPO: increased sensitivity and 1 day turn over | Existing test | Other services (HIV) |
| **Bonnet 2010 IJTLD** | Y | Bleach sedimentation microscopy | | N | N/A | N | Y | N | N | N | N | N/A | Existing test | Dx |
| **Dowdy 2003 JCM** | Y | Gen-Probe | | N | N/A | N | N | N | N | GenProbe | N | N/A | Existing test | Dx Rx |
| **Dowdy 2008 IJTLD** | Y | HYPO: Pont of Care Dx | | N | N/A | N | Y | N | N | N | Solid | HYPO: POC test | Product profile | Dx Rx |
| **Dowdy 2008 Plos ONE** | Y | Culture as first line | | N | N/A | N | Y | N | N | N | Liquid & Solid | N/A | Scale-up | Dx Rx |
| **Dowdy 2011 PLosMed** | Y | TB serology tests (anda-tb ELISA) | | N | N/A | N | Y | N | N | N | Liquid | Serological tests | Existing test | Dx Rx |
| **Guerra 2008 JCM** | Y | Gen-Probe (with sample dilution) | | N | N/A | N | Y | N | N | GenProbe | N | N/A | Existing test | Dx |
| **Hughes 2012 RespMed** | Y | NAAT | | N | N/A | N | Y | N | N | Non-specific | Solid | N/A | Existing test | Dx Rx |
| **Jones 2001 AJRCCM** | Y | MMR screening | | N | N/A | Y | N | Y (MMR) | N | N | N | Y (TST) | Existing test | Dx Rx |
| **Langley 2012 HCMS** | Y | Xpert | | Y | Operational & transmission | N | Y  (ZN, LED) | Y | Y | N | Solid | N/A | Existing test | Dx Rx |
| **Lim 2000 Resp** | Y | Amplicor assay (PCR), or CT | | N | N/A | N | N | N | N | Amplicor, NAAT on BAL | N | CT | Existing test | Dx Rx |
| **Maheswaran 2011 PLosONE** | Y | CXR Screening | | N | N/A | Y | Y | Y | N | N | N | N/A | Existing test | Dx Rx |
| **Menzies 2012 PMed** | Y | Xpert | | N | N/A | N | Y | Y | Y | N | Y | N/A | Existing test / Scale-up | other services (HIV) |
| **Meyer-Rath 2012 PLosONE** | Y | Xpert | | N | N/A | N | Y | Y | Y | LPA | Liquid | non-specific DST | Scale-up | Dx Rx |
| **Rajalahti 2004 ERJ** | Y | Amplicor (PCR) | | N | N/A | N | Y | N | N | Amplicor | Liquid | CT | Existing test | Dx Rx |
| **Resch 2006 PLosMed** | Y | DST | | N | N/A | N | N | N | N | N | DST | N | Existing test | Dx Rx |
| **Samandari 2011 AJRCCM** | Y | CXR Screening | | N | N/A | Y | N | Y | N | N | N | N | Existing test | Dx Rx |
| **Schnippel 2013 SAMJ** | Y | Xpert | | N | N/A | N | Y | Y | Y | LPA | Liquid | DST (non-specific) | Existing test | Dx Rx |
| **Sun 2013 IJTLD** | Y | Urine LAM | | N | N/A | N | N | N | N | N | N | Urine LAM | Existing test | Dx Rx |
| **Vassall 2011 PLosMed** | Y | Xpert | | N | N/A | N | Y | Y | Y | LPA | Liquid & Solid | conventional DST | Existing test | Dx Rx |
| **Winetsky 2012 PLoS Med** | Y | Xpert, mass miniature radiography (MMR) | | N | N/A | Y | Y | Y (MMR) | Y | Sputum PCR with probes for MDR | Liquid & Solid | N/A | Existing test | Dx Rx |

**Table S3.4:** Modeling methods

| **Reference** | **Data fit** | **Sensitivity** | **Pre-diag inf** | **False +** | **False -** | **Repeat entry** | | **Drug Susc.** | **HIV** | **Previous Treatment** |
| --- | --- | --- | --- | --- | --- | --- | --- | --- | --- | --- |
| **Abimbola 2012 AIDS** | N | one-way | N | N | N | N | N/A | N | Y | N |
| **Acuna CID 2008** | N | multi | N | Y | Y | N | N/A | Y | N | N |
| **Albert 2004 IJTLD** | N | one-way | N | Y | Y | N | N/A | N | N | N |
| **Andrews 2012 AIDS** | N | two-way | N | Y | Y | N | N/A | Y | Y | Y |
| **Bonnet 2010 IJTLD** | N | one-way | N | N | N | N | N/A | N | N | N |
| **Dowdy 2003 JCM** | N | multi | N | Y | N | N | N/A | N | N | N |
| **Dowdy 2008 IJTLD** | N | multi | N | N | N | N | N/A | N | Y | N |
| **Dowdy 2008 PlosONE** | N | multi | N | Y | Y | Y | Same | N | Y | N |
| **Dowdy 2011 PLosMED** | N | two-way | N | Y | Y | N | N/A | N | Y | N |
| **Guerra 2008 JCM** | N | one-way | N | N | N | N | N/A | N | N | N |
| **Hughes 2012 RespMed** | N | one-way | N | Y | Y | N | N/A | Y | N | N |
| **Jones 2001 AJRCCM** | N | One-way | N | N | N | N | N/A | Y | Y | N |
| **Langley 2012 HCMS** | N | one-way | Y | Y | Y | Y | Same | Y | Y | Y |
| **Lim 2000 Resp** | N | one-way | N | Y | Y | N | N/A | N | N | N |
| **Maheswaran 2011 PLosONE** | N | multi | N | Y | Y | Y | False Neg. | N | Y | N |
| **Menzies 2012 PlosMed** | Y | multi | Y | Y | Y | N | N/A | Y | Y | Y |
| **Meyer-Rath 2012 Plos ONE** | N | one-way | N | N | N | N | N/A | Y | Y | Y |
| **Rajalahti 2004 ERJ** | N | two-way | N | Y | N | N | N/A | Y | N | N |
| **Resch 2006 PLosMed** | Y | two-way | N | N | N | N | N/A | Y | N | Y |
| **Samandari 2011 AJRCCM** | Y | Multi | N | Y | Y | N | N/A | Y | N | N |
| **Schnippel 2013 SAMJ** | N | one-way | N | N | Y | N | N/A | Y | Y | N |
| **Sun 2013 IJTLD** | N | multi | N | Y | N | N | N/A | N | Y | N |
| **Vassall 2011 PLosMED** | N | multi | N | N | Y | N | N/A | Y | Y | Y |
| **Winetsky 2012 PLosMed** | Y | multi | Y | N | Y | N | N/A | N | Y | Y |
